# Deep learning-based pelvimetry in pelvic MRI volumes for pre-operative difficulty assessment of total mesorectal excision

**DOI:** 10.1007/s00464-024-11485-4

**Published:** 2025-01-03

**Authors:** Simon C. Baltus, Ritch T. J. Geitenbeek, Maike Frieben, Elina Thibeau-Sutre, Jelmer M. Wolterink, Can O. Tan, Matthijs C. Vermeulen, Esther C. J. Consten, Ivo A. M. J. Broeders

**Affiliations:** 1 Surgery Department, Meander Medical Centre, Maatweg, Amersfoort, 3818 TZ Utrecht The Netherlands; 2 Robotics and Mechatronics, University of Twente, Drienerlolaan, Enschede, 5722 NB Overijssel The Netherlands; 3 Surgery Department, University Medical Center Groningen, Hanzeplein, Groningen, 9713 GZ Groningen The Netherlands; 4 Department of Applied Mathematics, Technical Medicine Center, University of Twente, Drienerlolaan, Enschede, 5722 NB Overijssel The Netherlands

**Keywords:** Pelvimetry, Magnetic resonance imaging, Total mesorectal excision, U-Net

## Abstract

**Background:**

Specific pelvic bone dimensions have been identified as predictors of total mesorectal excision (TME) difficulty and outcomes. However, manual measurement of these dimensions (pelvimetry) is labor intensive and thus, anatomic criteria are not included in the pre-operative difficulty assessment. In this work, we propose an automated workflow for pelvimetry based on pre-operative magnetic resonance imaging (MRI) volumes.

**Methods:**

We implement a deep learning-based framework to measure the predictive pelvic dimensions automatically. A 3D U-Net takes a sagittal T2-weighted MRI volume as input and determines five anatomic landmark locations: promontorium, S3-vertebrae, coccyx, dorsal, and cranial part of the os pubis. The landmarks are used to quantify the lengths of the pelvic inlet, outlet, depth, and the angle of the sacrum. For the development of the network, we used MRI volumes from 1707 patients acquired in eight TME centers. The automated landmark localization and pelvic dimensions measurements are assessed by comparison with manual annotation.

**Results:**

A center-stratified fivefold cross-validation showed a mean landmark localization error of 5.6 mm. The inter-observer variation for manual annotation was 3.7 ± 8.4 mm. The automated dimension measurements had a Spearman correlation coefficient ranging between 0.7 and 0.87.

**Conclusion:**

To our knowledge, this is the first study to automate pelvimetry in MRI volumes using deep learning. Our framework can measure the pelvic dimensions with high accuracy, enabling the extraction of metrics that facilitate a pre-operative difficulty assessment of the TME.

## Introduction

Total mesorectal excision (TME) is the standard surgical treatment of rectal cancer, involving the precise dissection of the mesorectum [1]. There is a large variation in the difficulty of this procedure, depending on both clinical and anatomic factors [2, 3]. Among these, body mass index (BMI), sex, tumor location and size, and pelvic size are known predictive factors for difficulty and outcomes [4, 5].

During surgery, the bony pelvis directly constrains the surgical access to the rectum, which impacts the ability to perform dissections [6]. In particular, TME is challenging in patients with a narrow and deep pelvis, because the bony structure disturbs the surgical maneuver [7, 8]. However, the current definition of a technically challenging pelvis is based on the surgeon’s subjective clinical assessment, as anatomic criteria are often not quantified pre-operatively.

Pelvimetry, the radiologic measurement of the pelvic dimensions, has been implemented in different clinical fields [9, 10]. Several studies have shown an association between different pelvic measurements and surgical outcomes of the TME [6]. For example, a multivariate analysis by Zhou et al. showed that, among others, the length of the pelvic inlet and outlet were the main factors affecting the operating time [11]. Also, Kim et al. and Simpson et al. showed that pelvic depth and sacral angulation are predictors for the TME operating time and specimen quality [12, 13].

Although all patients undergo magnetic resonance imaging (MRI) for tumor staging, pre-operative radiologic measurement of the bony pelvis has not been incorporated into patient treatment. Manual measurements of the pelvis are labor intensive and time-consuming. Therefore, pelvimetry is not used as a predictor of difficult laparoscopic operations.

This study introduces a deep learning-based framework for automating the pre-operative measurement of the pelvic dimensions in MRI volumes. Our overarching objective is to create MRI-based metrics that facilitate a pre-operative difficulty assessment of the TME and, potentially, to improve surgical outcomes.

## Materials and methods

### Study population

The study used patients from the minimally invasive rectal carcinoma (MIRECA) cohort [14]. This cohort was part of a retrospective study performed in eleven institutes with a large group of patients comparing laparoscopic-TME versus robotic-TME versus transanal-TME for primary rectal cancer. Patients 18 years or older with MRI-defined rectal cancer (based on the sigmoidal take-off definition) who underwent tumor resection between January 2015 and December 2021 were included. Patients were enrolled for this study based on the availability of MRI scans, the presence of artifacts, and the field of view, which had to include the entire bony pelvis from the coccyx up to the sacral promontory.

The pre-operative MRI volumes were acquired using 1.5- and 3.0-Tesla (T) MRI scanners (Siemens Medical Solutions, Forchheim, Germany; Philips Medical Systems, Best, the Netherlands). 3D T2-weighted sagittal Turbo-spin-echo sequences from our institutional standardized abdominal pelvic MR imaging protocols were clinically used for pre-operative staging of the rectal tumor before surgery. These acquisitions were used for the automated measurement of the pelvic dimensions in our study.

### MRI pelvimetry

**Fig. 1:**
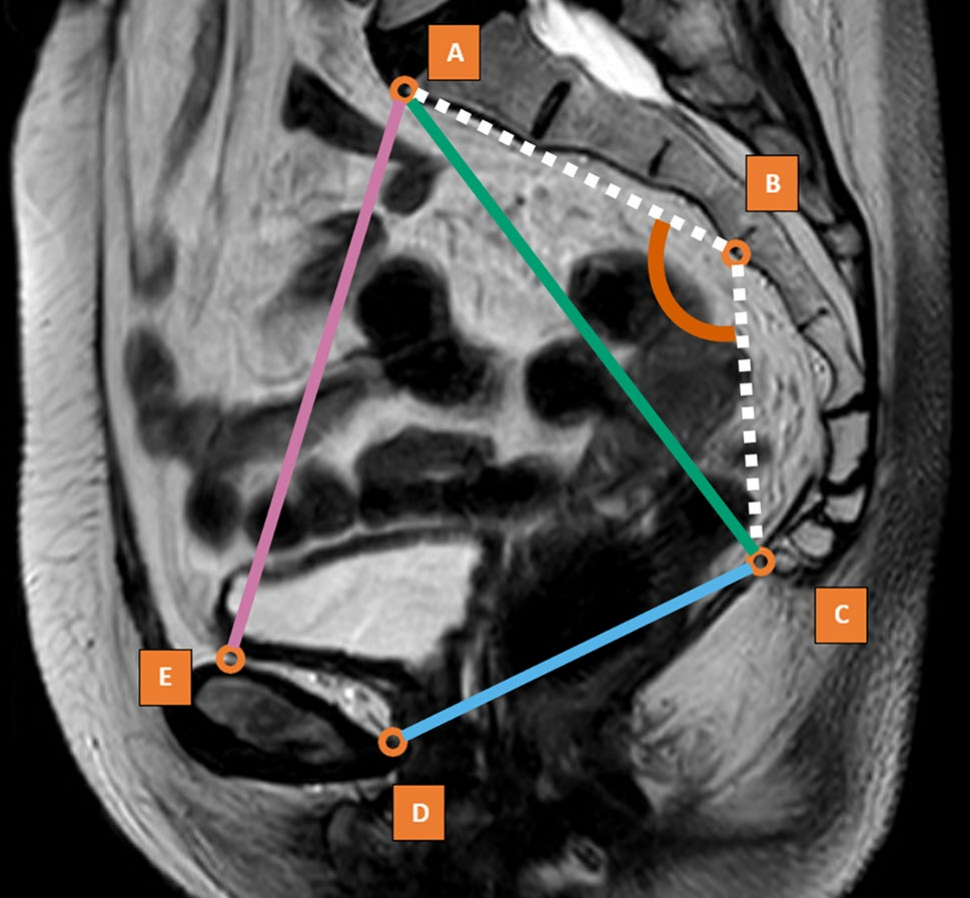
A visualization of the sagittal MRI acquisition with the five landmarks: promontorium (A), S3-vertebrae (B), coccyx (C), dorsal part of os pubis (D), and cranial part of os pubis (E). The landmarks are used to measure the pelvic inlet (purple, distance between A and E), pelvic outlet (blue, distance between C and D), pelvic depth (green, distance between A and C), and sacral angulation (orange, angle between A, B, and C) (Color figure online)

For this study, five anatomic landmarks visualized on clinical MRI volumes were manually annotated by two physicians trained by an abdominal radiologist. The landmarks comprised the promontorium (A), S3-vertebrae (B), coccyx (C), dorsal part of the os pubis (D), and cranial part of the os pubis (E). The five landmarks provide the basis for the measurements of lengths of the pelvic inlet, pelvic outlet, pelvic depth, and the angle of the sacrum (sacral angulation) (Fig. 1). These measurements have been suggested to be among the predictors for TME outcomes [6]. During the annotation of the landmarks, the midline on the sagittal MRI was used as the point of reference for annotation, which was identified visually by the most comprehensive display of the sacrum. The landmarks were recorded as 3D numerical coordinates and were used for the development and evaluation of the automated method.

### Model development

**Fig. 2:**
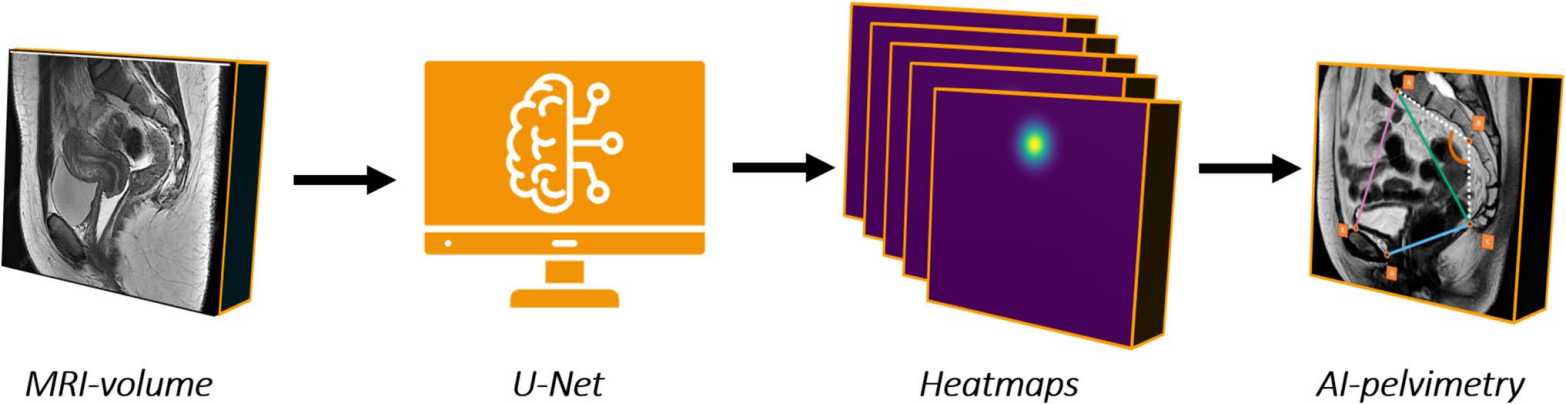
Visualization of the automated pelvimetry in pre-operative pelvic MRI volumes

As shown in Fig. 2, the measurements of the pelvic dimensions were automated using a deep learning framework. Similar to [15], we applied the 3D U-Net to regress heatmaps from input MRI volumes and therefore localize multiple landmarks [16]. Instead of using absolute landmark coordinates, a heatmap indicates the probability of the landmark location. After the development of the network, an MRI volume was used as input, and the network produced five heatmaps. The network output was converted to numerical coordinates by selecting the location of the maximum value in each heatmap, creating five distinctive landmark locations. The model was trained based on the manual annotation of the landmarks in the pre-operative MRI volumes [17].

MRI volumes were pre-processed by scaling each image such that its pixel size equaled the mean size among the whole dataset (0.67 × 0.67 × 4.3 mm). In addition, the intensity in each MR volume was normalized using a percentile-based normalization. The heatmap labels were generated based on the manual annotations combined with a Gaussian filter (Stand Deviation: 5 pixels). The model was trained end to end on the training dataset using the MSE-loss as the loss function, with a learning rate of 0.0001, a batch size of 32, and a patch size of 128 × 128 × 16 voxels. The model was trained over 500 epochs, with an early stopping if the validation loss did not improve for 50 epochs. The epoch with the lowest validation loss was chosen for model selection.

### Evaluation

A stratified fivefold cross-validation approach was used to evaluate the model’s performance. For each fold, 80% of the data from each institute was used to train the model, while the remaining 20% was reserved for testing. This stratification ensured that data distribution across training (*N* = 1365) and testing sets (*N* = 342) was consistent. By performing five distinct splits, we ensured every patient was included in the test set once, enabling assessments at the patient and institute levels. The Euclidean distance between the ground truth and model output on the test set was used to quantify the performance of the landmark localization. This localization error was evaluated using the mean absolute error (MAE). The difference in the measured dimensions computed between the ground truth and the predicted landmarks was also used for the performance assessment. For the pelvic inlet, outlet, and depth, the difference was measured by the Euclidean distance. For the sacral angulation, the difference in angle was used. The measurement error was also quantified by the MAE. The coefficient of determination (*R*^2^), Spearman correlation coefficient, and Bland–Altman plots were also used to quantify how well the predicted measurements approximate the manual data. The bias was calculated by the overall mean difference between the ground truth and predicted dimensions. In addition, the inter-observer variability of the landmark localization was computed using the MAE based on the blinded annotation of 500 patients by two annotators.

## Results

This study included 2292 patients from the MIRECA cohort. Of these, 248 patients did not have a pre-operative T2-weighted sagittal MRI acquisition. Based on visual inspection, 337 patients were further excluded due to the presence of imaging artifacts or incomplete depiction of the pelvic bones. Thus, MRI volumes from 1707 patients were used for the development of the model and the fivefold cross-validation.

### Automated localization of the pelvic landmarks

The fivefold cross-validation showed averaged over all the landmarks an MAE of 5.6 ± 8.4 mm. An overview of the landmark localization error for the individual landmarks and the institutes is provided in Table 1. The table shows that the landmarks are localized with a comparable MAE. The localization of landmark B, the S3-vertebrae, has both the largest mean error and standard deviation. The performance per institute showed an MAE varying between 4.7 and 6.2 mm. The MAE did not differ significantly between the 1.5- and 3.0-T acquisitions. The inter-observer variability between the two physicians was a MAE of 3.7 ± 8.4 mm.

**Table 1:** Localization error of landmarks (mm)

Institute	Landmark					Mean	*N* patients
	A	B	C	D	E		
1⋄,■	6.4 ± 10.5	6.8 ± 13.3	4.7 ± 11.1	5.1 ± 4.6	5.7 ± 3.5	5.7 ± 8.6	306
2♦,•,⋆,#	6.7 ± 9.5	6.7 ± 10.3	4.8 ± 5.9	5.7 ± 5.4	7.0 ± 8.3	6.2 ± 7.9	489
3□	5.4 ± 4.9	4.7 ± 4.4	3.7 ± 3.6	5.8 ± 4.3	5.5 ± 3.2	5.0 ± 4.1	65
4△,▲,#,◦	5.9 ± 5.9	7.5 ± 13.3	3.6 ± 3.0	6.1 ± 3.6	5.9 ± 3.8	5.8 ± 5.9	111
5□	5.1 ± 6.7	6.1 ± 12.7	4.0 ± 7.0	4.5 ± 7.5	6.1 ± 11.2	5.2 ± 9.0	421
6♦,•	4.4 ± 4.6	5.4 ± 10.8	4.1 ± 3.9	4.2 ± 2.6	5.6 ± 3.2	4.7 ± 5.0	80
7•	6.8 ± 7.4	4.3 ± 3.9	4.0 ± 3.9	3.6 ± 2.8	5.3 ± 3.2	4.8 ± 4.2	87
8■,◦	4.7 ± 5.3	7.5 ± 18.3	3.4 ± 3.0	4.7 ± 3.6	5.2 ± 3.1	5.1 ± 6.7	148
All	5.8 ± 8.2	6.4 ± 12.2	4.3 ± 6.9	5.1 ± 5.5	6.1 ± 7.5	5.6 ± 8.4	1707

The errors are quantified per institute (1–8) and per landmark (A-E). The landmarks are the promontorium (A), S3-vertebrae (B), coccyx (C), dorsal part of os pubis (D), and cranial part of os pubis (E). The used MRI scanners were as follows: ⋄: Interia, 1.5T, Philips Healthcare; ■: Ingenia, 1.5T, Philips Healthcare; ♦: Magnetom Aera, 1.5T, Siemens Healthineers; •: Magnetom Avanto, 1.5T, Siemens Healthineers; ⋆: Magnetom Sola, 1.5T, Siemens Healthineers; #: Magnetom Skyra, 3.0T, Siemens Healthineers; □: Achieva, 1.5T, Philips Healthcare; △: MAGNETOM Espree, 1.5T, Siemens Healthineers; ▲: MAGNETOM Symphony, 1.5T, Siemens Healthineers; ◦: MAGNETOM Verio, 3.0T, Siemens Healthineers

### Automated measurement of pelvic dimensions

**Fig. 3:**
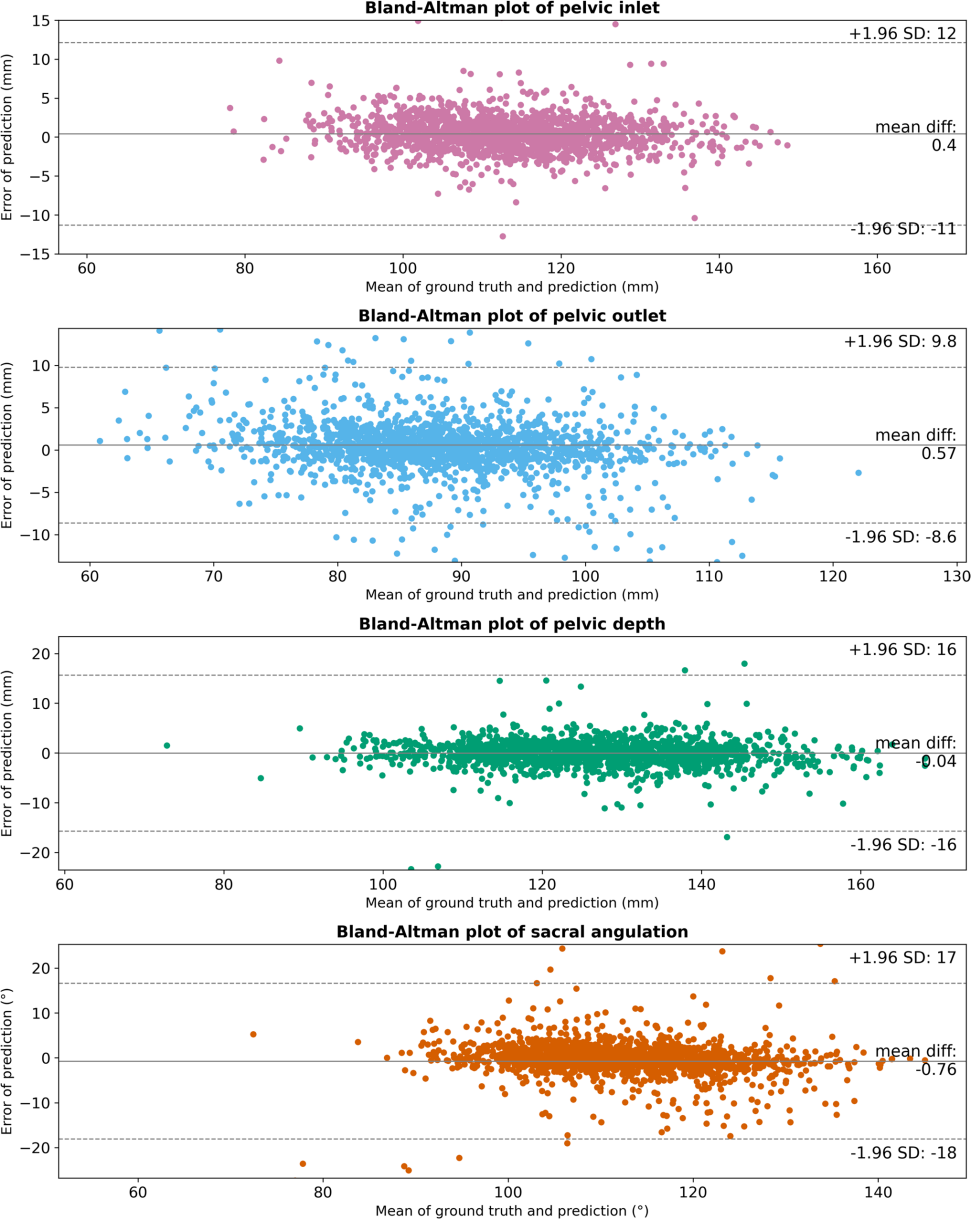
Bland–Altman plots for each of the pelvic dimensions: pelvic inlet (purple), pelvic outlet (blue), pelvic depth (green), and sacral angulation (orange) (Color figure online)

Table 2 provides an overview of the performance of the framework in measuring the different pelvic dimensions. Spearman correlation coefficient ranged between 0.7 and 0.87. In addition, excluding patients outside the limits of agreement (outliers) leads to an increase of *R*^2^, especially for the pelvic depth. Figure 3 shows the agreement between the predicted and manually measured pelvic dimensions. The figure indicates that there is no systematic bias for the measurements.

**Table 2:** Measurement error of the pelvic dimensions: pelvic inlet, pelvic outlet, pelvic depth, and sacral angulation

	Pelvic inlet	Pelvic outlet	Pelvic depth	Sacral angulation
MAE (mm/°)	2.0 ± 5.6	2.4 ± 4.1	2.8 ± 7.5	3.1 ± 8.3
*R*^2^	0.71	0.73	0.63	0.23
*R*^2^—without outliers	0.96	0.91	0.97	0.89
Spearman correlation coefficient	0.87	0.87	0.83	0.7
Limits of agreement (mm/°)	± 11.7	± 9.2	± 15.7	± 17.4
Bias (mm/°)	0.4	0.6	0	− 0.8

The error in the length of the pelvic inlet, outlet, and depth is measured in millimeters. The sacral angulation error is measured in degrees

## Discussion

In this work, we presented a deep learning-based workflow for automated pelvimetry in pre-operative MRI volumes. We showed that crucial landmarks can be localized with a mean error of 5.6 mm, which is comparable to the human inter-observer variability (3.7 ± 8.4 mm). In addition, these landmarks led to an accurate measurement of the clinically relevant pelvic dimensions. Therefore, the study results demonstrated that MRI-based metrics can be extracted automatically for difficulty assessment of the TME.

To our knowledge, this is the first study to automate pelvimetry in MRI volumes using deep learning. Due to the earlier absence of this automation, measuring these pelvic dimensions was not part of the clinical workflow. However, the dimensions provide insights into surgical access to the rectum and the associated challenges (e.g., disturbance of maneuver). Therefore, the proposed workflow can directly support the current practice as the given dimensions will enable physicians to make a more patient-specific assessment.

Several studies have explored the potential associations between pelvimetry and clinical outcomes in rectal cancer surgery, emphasizing the relevance of pelvic dimensions in surgical planning and prognosis. In previous studies, smaller pelvic dimensions, particularly the interspinous (IS) distance, intertubercle (IT) distance, and the pelvic inlet, have been associated with increased surgical difficulty and compromised outcomes. The retrospective analysis of the European MRI and rectal cancer surgery (EuMaRCS) study by d’Angelis et al. reported that a smaller IS was associated with higher conversion rates to open surgery [18]. Furthermore, Boyle et al. and Baik et al. reported that a smaller IS was associated with a positive circumferential margin [19, 20]. Similarly, reduced IT distances emerged as predictors of longer laparoscopic dissection times and increased post-operative complications in studies by Kim et al. and de’Angelis et al., highlighting its potential as an operative planning metric [12, 18]. Escal et al. demonstrated that an IT > 10.1 cm was associated with increased surgical difficulty according to their composite score [5]. Moreover, the pelvic inlet’s anterior–posterior dimension was related to critical surgical metrics, including CRM involvement, TME completeness, and intraoperative blood loss [8, 19, 20]*.* However, these compelling findings also reflect variability in the definitions of surgical difficulty and the pelvic measurement methods, which may influence the generalizability and interpretation of results.

Building on these findings, our study underscores the potential of automated pelvimetry on MRI as a standardized and efficient modality for pre-operative assessment of pelvic dimensions. This approach has implications for predicting surgical complexity and informing post-operative outcomes. By integrating such predictive measurements into pre-operative planning, clinicians could stratify patients based on risk to optimize surgical and perioperative strategies. In addition, it may potentially improve outcomes by tailoring interventions to individual anatomic challenges. However, our method requires further validation, and the associations with specific surgical outcomes need to be tested in prospective clinical studies to confirm its reliability in clinical practice. Also, it is essential to acknowledge that surgical complexity is inherently multifactorial, influenced not only by pelvic dimensions but also by patient- and tumor-specific factors, such as body mass index (BMI) and tumor height [21, 22]. Therefore, in addition to pelvic dimensions, a pre-operative assessment tool should also consider patient characteristics to make a comprehensive assessment. Further study will focus on applying machine learning to integrate both anatomic and clinical considerations. Such advancements can potentially support surgical planning, enhancing precision and patient-centered care.

**Fig. 4:**
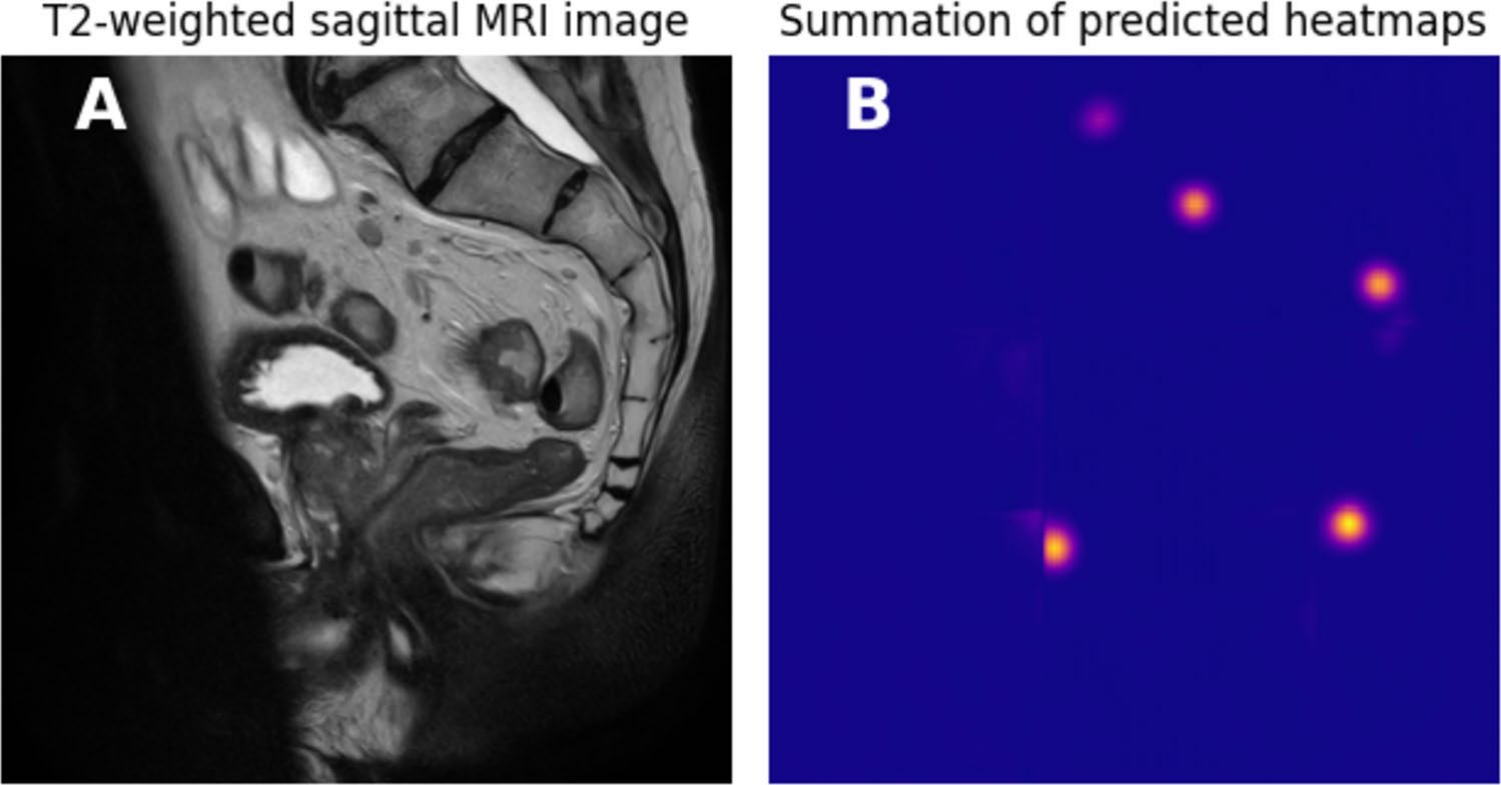
**A** An example visualization in which the banding artifact influences the depiction of the os pubis. **B** Summation of the predicted heatmaps, showing the inability of the model to localize the five landmarks

Notably, this study found that the performance of the automated extraction can be influenced by the imaging quality. For example, the LoA and *R*^2^ values with and without outliers (Table 2) indicated that the overall performance was affected by outliers. Visual inspection of the outliers showed that most outliers in the measurement of the pelvic inlet had an MRI acquisition presenting an MRI banding artifact. As shown in Fig. 4, the artifact partially covers the os pubis and is associated with a failure of the automated landmark detection. This indicated that the model cannot make an accurate pelvic dimension determination for every MRI acquisition. In exceptional cases, such as shown in Fig. 4, the resulting erroneous dimension measurement could influence the physician’s judgment. Therefore, clinical application of the method would require a visual check or an automated recognition of outliers. A method for recognizing outliers could be developed based on the uncertainty of the model tied to the predicted landmark location or the probability of the pelvic dimension. In case of low certainty within the landmark detection or an improbable measured dimension, this can be communicated to the practitioner or a manual measurement can be chosen.

Future research should also study the broader application of automated pelvimetry. First, an external validation can be performed on a prospective dataset. This would validate the performance of the methodology at other institutes. Secondly, the presented methodology should be studied on the automated measurement of other pelvic dimensions. For example, the methodology presented can be used in transverse MRI volumes. This would enable the automated measurement of the intraspinal and intratubular distances, which have also been described as predictive for TME outcomes [6]. Thirdly, the methodology can also be used for automated pelvimetry in CT acquisitions. A comparable performance for this modality would increase the applicability of the presented methodology. Finally, automated pelvimetry can be applied to detect landmarks relevant to other clinical fields. For instance, certain pelvic dimensions have been described in the literature as predictors for the radicality of the prostatectomy [18].

In conclusion, we showed that pelvimetry in MRI volumes can be automated using deep learning. The proposed workflow can extract pelvic dimensions with high accuracy, facilitating a more patient-specific assessment of the surgical difficulty. Future research will focus on quantifying the predictive value of the pelvic dimensions to create a pre-operative assessment tool for post-operative complications.
